# Novel therapies are changing treatment paradigms in metastatic prostate cancer

**DOI:** 10.1186/s13045-020-00978-z

**Published:** 2020-10-28

**Authors:** Eric Powers, Georgia Sofia Karachaliou, Chester Kao, Michael R. Harrison, Christopher J. Hoimes, Daniel J. George, Andrew J. Armstrong, Tian Zhang

**Affiliations:** 1 Department of Medicine, Duke University, Durham, NC 27710 USA; 2 Division of Medical Oncology, Department of Medicine, Duke University, DUMC 103861, Durham, NC 27710 USA; 3 Duke Cancer Institute Center for Prostate and Urologic Cancers, Durham, NC 27710 USA; 4 Department of Pharmacology and Cancer Biology, Duke University, Durham, NC 27710 USA

**Keywords:** Metastatic prostate cancer, Castration-resistant prostate cancer, Prostate-specific membrane antigen (PSMA), Polyadenosine diphosphate [ADP]-ribose polymerase (PARP) inhibitor, Androgen receptor inhibitors (ARIs)

## Abstract

Metastatic castration-resistant prostate cancer (mCRPC) remains a terminal diagnosis with an aggressive disease course despite currently approved therapeutics. The recent successful development of poly ADP-ribose polymerase (PARP) inhibitors for patients with mCRPC and mutations in DNA damage repair genes has added to the treatment armamentarium and improved personalized treatments for prostate cancer. Other promising therapeutic agents currently in clinical development include the radiotherapeutic 177-lutetium-prostate-specific membrane antigen (PSMA)-617 targeting PSMA-expressing prostate cancer and combinations of immunotherapy with currently effective treatment options for prostate cancer. Herein, we have highlighted the progress in systemic treatments for mCRPC and the promising agents currently in ongoing clinical trials.

## Introduction

Prostate cancer (PC) is the second most commonly diagnosed cancer among men worldwide, following lung cancer, and the first among men in the USA [1]. Although clinical outcomes are excellent for patients with localized disease, patients with metastatic prostate cancer (mPC) have poor prognosis, with a 5-year survival rate reaching 30%. Androgen deprivation therapy (ADT) has long been the treatment of choice as backbone of all other therapies, by reducing circulating androgens to castration levels and slowing the progression of the disease. Unfortunately, ADT as a single agent does not always prevent disease progression, and eventually hormone-sensitive prostate cancer (HSPC) will develop resistance even at low testosterone levels and become castration-resistant prostate cancer (CRPC).

Over the last few years, several successful phase-3 trials have expanded the available treatments in metastatic HSPC with docetaxel (CHAARTED, GETUG-AFU 15, and STAMPEDE [2, 3]), abiraterone acetate (STAMPEDE and LATITUDE), enzalutamide (ARCHES, ENZAMET [4, 5]), and apalutamide (TITAN [6]). In addition, current standard therapy for patients with CRPC apart from ADT includes sipuleucel-T, chemotherapy (docetaxel if no prior use, or cabazitaxel if prior docetaxel), abiraterone acetate, enzalutamide, olaparib and rucaparib (for molecularly selected patients with mutations in DNA damage repair genes), and radium-223 (for bone metastases). However, mCRPC remains a lethal diagnosis, and more effective therapeutic approaches against mCRPC are necessary to further improve clinical outcomes.

While progress is ongoing for many new targeted treatments in mCRPC, we will highlight here the clinical progress of the recently US FDA-approved poly ADP-ribose polymerase (PARP) inhibitors, new indications for the second-generation androgen receptor antagonists, and promising radiopharmaceutical and immunotherapy agents.

## Main text

### PARP inhibitors

Polyadenosine diphosphate [ADP]-ribose polymerase (PARP) is a nuclear enzyme that aids the repair of single-strand DNA breaks (SSBs) [7–9]. Cells with an intact repair apparatus have two major mechanisms for repairing double-strand breaks (DSBs), including homologous recombination repair (HRR) and non-homologous end-joining (NHEJ). Cells with mutations in the HRR machinery, such as those with mutations in *BRCA1, BRCA2*, and *ATM,* are forced toward the more error-prone DSB repair pathway of NHEJ, leading to genomic instability and cellular death. Based on this concept, the first application of PARP inhibitors was in patients with ovarian cancer; patients with *BRCA1/2* and other HRR mutations achieved longer progression-free survival (PFS) benefits [10–12].

In mCRPC, PARP inhibitors were first applied in those patients who harbored *BRCA1/2* mutations and had already progressed on previous treatments [13]. In a phase-2 clinical study, 49 patients with mCRPC were treated with olaparib. Sixteen out of these 49 patients had somatic or germline mutations in DNA repair genes. Eighty-eight percent (14/16) of patients with DNA repair gene mutations reached significantly longer PFS (9.8 vs. 2.7 months) and overall survival (OS, 13.8 vs. 7.5 months) compared to those patients without these mutations [14]. In a subsequent phase-2 study of 92 patients with DNA repair gene aberrations, patients were randomized to olaparib at either 300 mg or 400 mg twice daily. Of the 46 patients treated with 400 mg, 25 patients (54%) had an objective response (OR) and 18/46 (39%) patients in the 300 mg group had objective responses [15].

Recently published data from the open-label phase-3 PROfound trial (NCT02987543) confirmed the efficacy of olaparib in patients with mCRPC. Three hundred and eighty-seven patients with mCRPC progressing on prior abiraterone or enzalutamide were randomized 2:1 to receive either olaparib 300 mg twice daily or investigator’s choice of enzalutamide or abiraterone acetate. Patients were divided into two cohorts based on their HRR gene mutation. Patients with mutations in *BRCA1*, *BRCA2*, or *ATM* were randomized in cohort A (*N* = 245), and patients with mutations among 12 other genes involved in the HRR pathway were randomized in cohort B (*N* = 142). In patients with at least one alteration in *BRCA1/2* or *ATM* (cohort A), olaparib prolonged radiographic PFS (rPFS) from 3.6 to 7.4 months (HR = 0.34; 95% CI 0.25–0.47; *p *value < 0.001), and improved median OS from 15.1 months (control cohort) to 18.5 months (olaparib-treated cohort) (HR = 0.64, 95% CI 0.43–0.97, *p* = 0.02) [16]. In addition, the confirmed objective response rate (ORR) was 33% (28/84 patients) in the olaparib group and 2% (1/43 patients) in the control group (OR 20.86, 95% CI 4.18–379.18, *p* < 0.001). Fifty percentage of prostate-specific antigen (PSA) decline responses was confirmed in 43% (66/153) of patients in the olaparib group and 8% (6/77) in the control group. The observed efficacy not only in cohort A but also in the overall population was seen regardless of whether olaparib monotherapy was administered before or after chemotherapy. The most common grade 3 and higher adverse events from olaparib included anemia (21%), fatigue (3%), nausea/vomiting, dyspnea, and urinary tract infections (2% each) [16]. When evaluating subsets of patients with different HRR mutations, patients with *BRCA2* mutations tended to achieve better responses and longer rPFS than patients who had *BRCA1* or *ATM* mutations.

Based on these results, olaparib was fully approved by the US FDA in May 2020 for patients with mCRPC who have deleterious or suspected deleterious germline or somatic HRR gene mutations and whose cancer has progressed with abiraterone or enzalutamide. However, given that olaparib was not compared against chemotherapy, patient selection for olaparib should depend upon the mutation and whether a standard treatment (such as chemotherapy) might be an available, potentially more active treatment.

In a similar approach, the TRITON2 study led to an accelerated FDA approval of rucaparib 600 mg twice daily for patients with mCRPC, *BRCA1/2* mutations and prior progression from both androgen receptor-directed treatment and taxane-based chemotherapy. The TRITON2 (NCT02952534) study was a multicenter, single-arm trial of 190 patients with *BRCA1/2, ATM* or other prespecified *DDR*–mutated mCRPC who had disease progression on prior androgen receptor-directed therapy and taxane-based chemotherapy [17]. Among patients with a *BRCA1/2* alteration and measurable disease at baseline, the ORR was 43.9% (95% CI 30.7–57.6). Moreover, 59.6% (34/57) of patients achieved a confirmed PSA response (≥ 50%) (95% CI 45.8–72.4), and the median duration of PSA response was 6.5 months (95% CI 5.7–7.5). The most common any grade adverse events (AEs) in rucaparib-treated patients included asthenia/fatigue (55.3%), nausea (49.5%), anemia (37.9%), and decreased appetite (27.9%). The confirmatory phase-3 TRITON3 trial continues to enroll and randomize patients with mCRPC and mutations in *BRCA1/2* or *ATM* to rucaparib versus physician’s choice of therapy (NCT02975934).

### Early evidence regarding combinations of PARP inhibitors with standard mCRPC therapies

There is conflicting evidence supporting the use of PARP inhibitors in mCRPC patients without mutations in DNA repair genes. The potential utility of PARP inhibitors in this setting will likely only be in combination with another effective agent. Preclinical studies have shown that inhibiting the androgen pathway can induce cell sensitivity to PARP inhibition, suggesting a synergy between androgen pathway blockade and PARP inhibitors—forming the hypothesis of multiple clinical trials [18–20]. Ongoing phase 2/3 controlled clinical trials investigating PARP inhibitors in mCRPC with or without the concurrent administration of another agent have been summarized (Table 1).

**Table 1:** Ongoing phase 2/3 controlled trials investigating PARP inhibitors in mCRPC

Trial name	Intervention	Control	DNA repair mutation required	Prior treatment for mCRPC allowed	Estimated enrollment (patients)	Trial phase	Clinicaltrials.gov identifier
BRCAAway	Olaparib or olaparib/AAP	AAP	Yes	No	70	2	NCT03012321
PROpel	Olaparib/AAP	AAP	No	No	720	3	NCT03732820
COMRADE	Olaparib/Ra 223	Ra 223	No	Yes	112	1/2	NCT03317392
KEYLYNK-010	Olaparib/pembrolizumab	AAP or enzalutamide	No	Yes	780	3	NCT03834519
KEYNOTE-365	Olaparib/pembrolizumab	Pembrolizumab + one of docetaxel, enzalutamide, or AAP	No	Yes	400	1b/2	NCT02861573
TRITON3	Rucaparib	AAP or enzalutamide or docetaxel	Yes	No	400	3	NCT02975934
N/A	Rucaparib or rucaparib/nivolumab	Nivolumab	No	No	60	1b/2	NCT03572478
CheckMate 9KD	Rucaparib/nivolumab	Nivolumab + enzalutamide or docetaxel	No	Yes	330	2	NCT03338790
MAGNITUDE	Niraparib/AAP	AAP	No	No	1000	3	NCT03748641
TALAPRO-2	Talazoparib/enzalutamide	Enzalutamide	No	No	1037	3	NCT03395197

mCRPC: metastatic castration-resistant prostate cancer; AAP: abiraterone and prednisone; Ra 223: radium 223 dichloride; VEGF: vascular endothelial growth factor

A phase-2 randomized trial of 142 patients comparing olaparib with abiraterone acetate to placebo with abiraterone acetate found a significant difference in rPFS (13.8 vs. 8.2 months, respectively, *p* = 0.034) [19]. The presence of DNA damage repair gene alterations was not an inclusion criterion, although a prespecified subgroup analysis of those patients with pertinent mutations (21/142) found no difference in PFS between the treatment cohorts, though the small numbers limited the subset’s power to detect a difference. A separate phase-2 trial of 148 patients compared veliparib plus abiraterone acetate to abiraterone acetate alone and found no difference in either PSA reduction or radiologic response, although secondary analysis showed a signal for improved outcomes in the small subgroup of patients with DNA repair defects [20]. The difference in these two trials may be explained by the reduced potency of veliparib compared to olaparib to trap PARP on single-strand breaks [21]. The phase-3 PROpel trial (NCT03732820) investigating olaparib plus abiraterone acetate in patients who have not yet received chemotherapy or anti-androgen therapy has completed enrollment and will provide further clarity on this issue. In addition to anti-androgen agents, combinations of PARP inhibitors with other treatments for prostate cancer (such as anti-angiogenic, radioligand, and immunotherapy) are being investigated in ongoing trials [22].

### Novel androgen receptor inhibitors: new indications in non-metastatic CRPC

Androgen receptor (AR) signaling remains an important driver of tumor growth even in CRPC [23]. Second-generation AR antagonists such as enzalutamide have become standard of care for CRPC [24]. These agents have greater affinity and no agonist activity to the AR binding domain, thereby blocking the nuclear translocation of AR and decreasing downstream androgen-dependent genes [25]. Enzalutamide significantly improved OS and PFS in mCRPC compared to placebo in phase-3 trials, both in the chemotherapy-pretreated (**AFFIRM** [26]) and in the chemotherapy-naïve settings (**PREVAIL** [27]).

For patients with non-metastatic CRPC (nmCRPC), recent data from the double-blinded, phase-3 **PROSPER** trial showed that administration of enzalutamide plus ADT prolonged median OS to 67.0 months compared to 56.3 months for the placebo plus ADT group (HR 0.73, 95% CI 0.61–0.89; *p* = 0.001). Fatigue and musculoskeletal events were the most frequent adverse events [28].

In addition, apalutamide and darolutamide have both gained US FDA approval in nmCRPC, based on phase-3 trials showing prolongation of metastasis-free survival (MFS). In the **SPARTAN** study of apalutamide versus placebo, MFS was prolonged by two years in the apalutamide cohort (40.5 vs. 16.2 months), while median OS was not yet reached in the apalutamide cohort versus 39 months in the placebo cohort [29, 30].

In the phase-3 **ARAMIS** trial, 1509 patients with nmCRPC were randomized to ADT plus either darolutamide or placebo [31]. The final analysis showed a statistically significant OS benefit corresponding to a 31% reduction in the risk of death in the treatment cohort (HR 0.69, 95% CI 0.53–0.88, *p* = 0.003) [32]. Regarding the most common AEs of darolutamide (any grade), only fatigue (12.1% vs. 8.7%), back pain (8.8% vs. 9.0%), arthralgia (8.1% vs. 9.2%), and hypertension (6.6% vs. 5.2%) were different between the two groups. There were no differences in seizures (0.2% for both groups), fractures (4.2% vs. 3.6%), or falls (4.2% vs. 4.7%) noted between the two groups.

It is clear that these agents provide statistically significant and clinically meaningful benefit in the treatment of nmCRPC. Darolutamide appears to have a lower rate of AEs [33]. Given its favorable toxicity profile, darolutamide may emerge as the agent of choice for patients who are on neuroactive medications or otherwise at increased risk for neurologic AEs. The ongoing phase-2 trials **ODENZA** and **ARACOG** comparing darolutamide and enzalutamide in mCRPC will address outcomes of patient preference and cognitive function, respectively (NCT03314324, NCT04335682). Although the OS data from PROSPER, SPARTAN, and ARAMIS are not mature (longer follow-up is required), studies have found a strong association of MFS with OS, as well as quality-of-life measures and PSA progression, making MFS a clinically important surrogate endpoint [34, 35], leading to the approval of these three AR antagonists in nmCRPC.

A novel treatment targeting degradation of the androgen receptor has emerged as an alternative potential therapeutic approach in patients with mCRPC. In particular, proteolysis-targeting chimeras (PROTACs) are heterobifunctional molecules that work by creating a trimeric complex between a target protein and an E3 ubiquitin ligase, facilitating target ubiquitination and subsequent degradation [36, 37]. Recently published data suggested that ARCC-4, a low-nanomolar AR degrader, is able to degrade about 95% of cellular AR [37]. Moreover, ARCC-4 inhibits prostate tumor cell proliferation even in high androgen environments and degrades clinically relevant AR with point mutations resistant to enzalutamide, addressing enzalutamide-resistant hurdles [37, 38]. Additionally, in enzalutamide-resistant model systems, administration of another AR degrader, ARD-61, in vitro and in vivo results has shown more potent anti-proliferative, pro-apoptotic effects [39]. The first phase-1 trial of ARV-110, an orally bioavailable PROTAC, in 18 patients with mCRPC, showed that two patients achieved confirmed ≥ 50% PSA reduction (both treated with ARV-110 at 140 mg once daily). There were two patients who developed grade 3/4 elevated AST/ALT levels but no other observed grade 3 or 4 treatment-related AEs [40].

While the second-generation AR inhibitors now have an expanded role in nmCRPC, ongoing clinical development of AR degraders and other novel therapies may expand treatment options in the future. In addition, it should be noted that these AR inhibitors all bind AR in the testosterone-binding domain, and therefore, resistance mechanisms may be similar and the agents likely would not have further efficacy if used as sequential monotherapies.

### Radiopharmaceuticals

Radiopharmaceutical agents allow systemic delivery of radiotherapy. In prostate cancer, phosphorus-32, strontium-89, and samarium-153 have been studied but did not show a survival benefit [41, 42]. Phosphorus-32 (^32^P), a β-emitter, the first US FDA-approved radiopharmaceutical in 1952, localizes to remodeling areas in bone including osteoblastic lesions and can relieve cancer-related bone pain [43–45]. Strontium-89 (^89^Sr), a β-particle emitter that functions in vivo as a calcium analog, was FDA-approved for management of bone metastatic CRPC in 1993; a phase-3 trial in bone metastatic CRPC demonstrated improvement in palliation of bone pain but with no survival benefit [44, 46]. Samarium-153 (^153^Sm), a β- and γ-emitter, was FDA-approved in 1997 as a chelate with ethylenediaminetetramethylenephosphonic acid (Sm-EDTMP or ^153^Sm lexidronam), which interacts with hydroxyapatite of bone in regions of osteoblastic lesions; the FDA approval was based on phase-3 studies demonstrating pain palliation, but no survival benefit was detected [44, 45, 47, 48].

Radium-223 is currently the only radiopharmaceutical treatment for mCRPC that improved overall survival for patients with mCRPC and symptomatic bone metastatic disease [49–51]. More recently, two other radioisotopes, lutetium-177 (^177^Lu) and gallium-68 (^68^ Ga), are being developed for patients with mCRPC and targeted to the cell surface molecule, prostate-specific membrane antigen (PSMA). While ^68^ Ga is mainly being developed as an imaging agent in positron emission tomography (PET) scans, ^177^Lu is the main therapeutic radioisotope and will be the focus of discussion here.

### 177-Lutetium-PSMA-617

^177^Lutetium (^177^Lu) is a beta-emitting, medium-energy radioisotope that is ideally suited for use in mCRPC due to its desirable physical properties: (1) the maximum energy β-emission of 0.5 meV with short penetration range of around 0.67 mm, delivering radiotherapy even to small-volume tumors, (2) the long half-life of ~ 7 days that prolongs its anti-tumor effect, and 3) the short particle range of 1.5 mm, limiting its cytotoxicity to the target tissue [52]. According to preclinical studies, ^177^Lu-DOTA-PSMA-617 (^177^Lu-PSMA), a PSMA-targeted small molecule, had shown high uptake and retention in mPC cells, and lower uptake in normal PSMA-expressing cells, such as in the kidney [53, 54]. This selective property and recent clinical activity render ^177^Lu-PSMA an exciting radioligand currently in clinical development for mPC [55]. In a single-arm phase-2 trial investigating ^177^Lu-PSMA in 30 patients with mCRPC who had progressed despite extensive prior therapy [56], 17/30 (57%) had > 50% PSA decline, and 14/17 (82%) patients with measurable disease had an OR. These data were reinforced in a follow-up analysis of the same cohort (with 20 additional patients) on later follow-up, with 64% of patients experiencing > 50% PSA decline. In patients treated with ^177^Lu-PSMA, median PSA PFS was 6.9 months (95% CI 6.0–8.7) [57]. Patients predominantly had disease progression in the bone marrow and in the liver. The two most common reasons for treatment discontinuation of ^177^Lu-PSMA were leukoerythroblastic pancytopenia and liver metastases [58]. The observed preliminary clinical efficacy prompted two larger randomized controlled trials (RCTs): a phase-2 trial randomizing 200 patients to either ^177^Lu-PSMA or cabazitaxel (**TheraP**-NCT03392428) and a phase-3 trial randomizing 750 patients to ^177^Lu-PSMA plus standard of care or standard care alone (**VISION**-NCT03511664). The **TheraP** study selected patients with PSMA-positive mCRPC progressing after docetaxel. The trial treated 98 patients with ^177^Lu-PSMA and 85 patients with standard-of-care cabazitaxel. Recently presented data from this study showed that 66% of patients treated with ^177^Lu-PSMA vs. 37% of patients treated with cabazitaxel achieved the primary endpoint of ≥ 50% PSA decline, a 29% absolute increase in the ≥ 50% PSA decline response (95% CI 16–42%; *p* < 0.0001). In addition, at a median follow-up of 13.3 months, ^177^Lu-PSMA was shown to significantly improve PSA PFS (HR 0.69, 95% CI 0.50–0.95; *p* = 0.02) [59]. Patients treated with ^177^Lu-PSMA also had fewer grade 3/4 AEs, most common of which were thrombocytopenia (11%), anemia (8%), and fatigue (5%). These results suggest that ^177^Lu-PSMA represents an effective therapy in patients with mCRPC and high PSMA expression. Further clinical development of ^177^Lu-PSMA is ongoing; the **VISION** study has completed enrollment of 750 patients and will subsequently report on its primary endpoint of overall survival [60]. This trial will pave the registrational path for potential approval from the US FDA for ^177^Lu-PSMA [55, 61].

### Immunotherapy

In recent years, immunotherapy has emerged as a treatment option for many malignancies with beneficial and durable responses [62–64]. The central principle is to enhance the anti-tumor activity of CD8 + cytotoxic T lymphocytes (CTLs), either by stimulating their activation against tumor-associated antigens (TAAs) or by blocking the immune-suppressing signals that decrease the number and exhaust the cytotoxic function of CTLs.

### Tumor-associated antigen-directed therapies

Multiple methods have been utilized to induce a T cell response against TAA-expressing tumor cells [64]. Monoclonal antibodies (mAbs) directly target a TAA, marking the tumor cell for destruction via multiple pathways: (1) activation of the complement system, (2) antibody-dependent cytotoxic T cell activation, or (3) enhancing uptake by phagocytes, followed by presentation to immature T cells. Vaccination is an alternative method to trigger immune response, either by direct administration of an antigen along with costimulatory molecules, or by stimulating a patient’s leukapheresed immune cells with an antigen ex vivo and reinfusing the cells. The above mechanism was the basis for sipuleucel-T, the first vaccine-based FDA-approved cancer therapy, which prolonged OS in mCRPC [65]. Interestingly, a recently published analysis of a registry of patients who received ≥ 1 sipuleucel-T infusion showed an OS difference between African-American and Caucasian patients in both the all-patient set ((HR 0.81, 95% CI 0.68–0.97, *p* = 0.03) and the PSA-matched patient subset (HR 0.70, 95% CI 0.57–0.86, *p* < 0.001). In particular, with a median follow-up of 46.6 months, the median OS for African-American patients was 35.3 versus 25.8 months for Caucasian patients, and this difference was greater, 54.3 versus 33.4 months, respectively, in patients who were treated at lower baseline PSA levels (HR 0.52, 95% CI 0.37–0.72, *p* < 0.001) [66].

A third TAA-directed modality involves inducing the patient’s own dendritic cells (DCs) to generate a T cell response against TAAs, either by loading them with antigen ex vivo or by genetically modifying them to present TAA. Table 2 presents TAAs specific to mPC that have been identified as targets for vaccines in mCRPC.

**Table 2:** Tumor-associated antigens (TAAs) in mPC immunotherapeutics

TAA	Function	Modalities for immunotherapy	Other expressing tissues	Unsuccessful therapies	Approved therapies	Ongoing trials
PSMA	Zinc metalloenzyme	Bi-specific antibodies, CAR-T, STEAP-1	Salivary glands, kidney	N/A	N/A	PSMAxCD3 antibody CC-1 (NCT04104607), P-PSMA-101 CAR-T (NCT04249947), PSCA-CAR-T (NCT03873805), CART-PSMA-TGFβRDN (NCT04227275), BPX-601 CAR-T (NCT02744287), AMG 509 (NCT04221542), Pasotuxizumab (BAY 2010112) (NCT01723475), ES414 (NCT02262910), Adoptive transfer of autologous T cells (NCT01140373)
	Folate uptake					
PAP	Seminal fluid production	Vaccine + ICI	Not significant	N/A	Sipuleucel-T	NCT04090528, NCT02499835
PSA	Serine protease	Vaccine + ICI	Not significant	Vaccine (NCT01322490)	N/A	NCT02933255, NCT02325557
	Forms semen coagulum					
MUC1	Cell adhesion, intracellular signaling	Vaccine, DC vaccine	Most epithelial cells. Many adenocarcinomas. Not expressed by normal prostate cells	N/A	N/A	NCT03481816
PSCA	Unknown	Vaccine, mAb	Not significant	N/A	N/A	N/A
TARP	Androgen regulation. Mitochondrial lipid metabolism	DC vaccine	Breast adenocarcinoma	N/A	N/A	NCT02362451

*PSMA* prostate-specific membrane antigen, *PAP* prostate acid phosphatase, *PSA* prostate-specific antigen, *MUC1* mucin-1, *PSCA* prostate stem cell antigen, *STEAP-1* six transmembrane epithelial antigen of the prostate 1, *TARP* T cell receptor gamma chain alternate reading frame protein, *CAR-T* chimeric antigen receptor T cell, *ICI* immune checkpoint inhibitor therapy, *DC* dendritic cell, *mAb* monoclonal antibody

Despite previous promising data from a phase-2 clinical trial on identifying other TAA-directed targets, two phase-3 clinical trials have failed to meet their clinical endpoints: **PROSTVAC** (targeting PSA) and **GVAX** (cellular vaccine with two irradiated prostate cancer cell lines) [67, 68]. Currently, concurrent administration of DCVAV with standard chemotherapy (docetaxel) is under investigation in a randomized, double-blinded, multicenter phase-3 study (**VIABLE**, NCT02111577).

### Immune checkpoint inhibitor therapy

Immune checkpoint inhibitor (ICI) therapy has shown clinical benefit in a number of solid tumors (e.g., metastatic melanoma, non-small cell lung cancer, renal cell carcinoma, and urothelial cancer, among others), but unfortunately these observations have not been replicated in patients with mCRPC [69, 70]. Factors such as low tumor mutational burden (TMB), loss of tumor suppressors (such as PTEN), low prevalence of DDR genetic defects, and silencing of major histocompatibility complex-1 (MHC-1) expression may all contribute to mCRPC’s relative lack of response to ICI therapy [71]. Two early phase-3 studies of the anti-cytotoxic T lymphocyte-associated protein-4 (CTLA-4) antibody ipilimumab both failed to meet their primary endpoint of improved OS [69, 70]; however, recent studies investigating the efficacy of the programmed death-1 inhibitor (PD-1) pembrolizumab have shown promising responses in patients with mCRPC. In a single-site cohort of 48 patients with mCRPC treated with pembrolizumab, 17% had ≥ 50% PSA decline with 8% (4/48 patients) having ≥ 90% PSA decline as best response [72]. These exceptional responders were found to have molecular changes (microsatellite instability-high (MSI-H), TMB-high, and mutation in *LRP1b*), which predispose to anti-PD-1 responses.

In the phase-2 KEYNOTE-199 study, patients with mCRPC were enrolled into one of five cohorts based on their measurable disease status, tumor PD-L1 status (by combined positive score, CPS), and prior enzalutamide experience and assigned to treatment with pembrolizumab monotherapy (cohorts 1–3 [73]) or pembrolizumab with enzalutamide (cohorts 4 and 5 [74]). In the pembrolizumab monotherapy cohorts, 133 patients with RECIST-measurable, PD-L1-positive mCRPC were enrolled in cohort 1, 66 patients with RECIST-measurable, PD-L1-negative mCRPC in cohort 2, and 59 patients with bone-predominant mCRPC in cohort 3. Biochemical response occurred in 23% of patients, with PSA stability in 9%. ≥ 50% PSA decline (PSA50) was noted in 9%, and 5% experienced ≥ 90% PSA reduction. The ORR was 5% (95% CI 2–11%) in cohort 1 and 3% (95% CI < 1% to 11%) in cohort 2. Interesting data emerged for duration of response (DOR) in this population of patients with those in cohort 1 having a median DOR that was not reached (range 1.9 to ≥ 21.8 months), while those in cohort 2 had a median DOR of 10.6 months (range 4.4–16.8 months). The median rPFS was 2.1 months (95% CI 2.0–2.1 months), 2.1 months (95% CI 2.0–3.3 months), and 3.7 months (95% CI 2.1–4.2 months) in cohorts 1, 2, and 3, respectively. Moreover, in cohort 1, the median OS was 9.5 months (95% CI 6.4–11.9 months), in cohort 2 was 7.9 months (95% CI 5.9–10.2 months), and in cohort 3 was 14.1 months (95% CI 10.8–17.6 months), while the estimated 12-month survival rates were 41%, 35%, and 62%, respectively. Up to 60% of the patients in this study experienced at least one treatment-related AE, and the most common AEs were fatigue, diarrhea, and decreased appetite. Also, one or more grade 3–5 treatment-related AEs were identified in 15% of these patients, 5% discontinued the treatment due to AEs, and two patients died due to pneumonitis and sepsis that were considered as treatment-related AEs [73].

While pembrolizumab monotherapy demonstrated promising responses in patients with mCRPC based on their durability, objective response rates were still low (3–5%) and therefore pembrolizumab was combined with enzalutamide cohorts 4 and 5 of Keynote 199. A previous single-institution study of concurrent pembrolizumab with enzalutamide in 10 patients with mCRPC, who had previously progressed on enzalutamide monotherapy, found that the combination can elicit regained and durable responses [75]. KEYNOTE-199 enrolled 81 men in cohort 4 and 45 men in cohort 5. All men had prior progression of disease on enzalutamide, and pembrolizumab was added to enzalutamide as further treatment. In cohort 4, the ORR was 12% including 2% complete responses, and 51% of patients achieved disease control in both the measurable (cohort 4) and bone-only metastatic (cohort 5) populations [74].

Other pembrolizumab combinations are in clinical development to improve the efficacy of known effective treatments for mCRPC. A phase 1b/2 trial (KEYNOTE-365) investigated the efficacy and safety of the concurrent pembrolizumab with olaparib in patients with mCRPC who previously progressed while on docetaxel. The updated results reported that 9% achieved PSA responses, and the median time to PSA progression was 16 weeks (95% CI 12–19). The median rPFS and OS were 4 months (95% CI 3–8) and 14 months (95% CI 8–19), respectively. Any treatment-related AEs occurred in 70 (83%) patients, including most commonly nausea (33%) and anemia (31%), as well as grade 3–5 AEs in 29 (35%) patients [76].

Since May 2019, an ongoing phase-3 trial (KEYLYNK-010) is currently enrolling patients to compare the combination of pembrolizumab and olaparib with investigator’s choice of either enzalutamide or abiraterone acetate. The primary endpoints of the study include rPFS and OS [77]. Another ongoing phase-3 trial (KEYNOTE-921) is investigating the combination of pembrolizumab and docetaxel in chemotherapy-naïve mCRPC patients, who have already progressed while on enzalutamide or abiraterone. The primary endpoints of this study include rPFS and OS [78]. A third randomized, double-blind phase-3 clinical trial (KEYNOTE-991), with an estimated number of participants above 1,200, is also accruing patients to the treatment of pembrolizumab with enzalutamide and ADT compared to enzalutamide and ADT in patients with mHSPC. The completion of these three phase-3 trials may expand treatment options for patients with mPC to include pembrolizumab combinations.

Another PD-1 inhibitor, nivolumab, has also been studied in combination with docetaxel, rucaparib, or enzalutamide in a phase-2 trial (CHECKMATE-9KD) for patients with mCRPC. In 41 patients treated with nivolumab and docetaxel, the ORR was 36.8%, with 1 complete and 6 partial responses. In addition, the confirmed PSA response was 46.3% (95% CI 30.7–62.6%), the median rPFS was 8.2 months (95% CI 6.6–not reached), and the 6-month rPFS rate was 71.5%. Any grade AEs occurred in almost all (92.7%) patients, and grade 3/4 AEs occurred in 48.8% of patients [79].

Finally, a phase-3 trial (IMbassador250) compared the anti-PD-L1 therapy atezolizumab with enzalutamide with enzalutamide alone in 759 patients with mCRPC. No difference was found in disease control between the two cohorts. In particular, the reported rPFS was 4.2 months (4.1–5.3) for combination of atezolizumab and enzalutamide versus 4.1 months for enzalutamide alone (3.7–4.5) (HR 0.90, 95% CI 0.75–1.07, *p* = 0.24), and time to PSA progression was 2.8 months versus 2.8 months (HR 1.04 95% CI 0.87–1.24, *p* = 0.6857). Median OS did not differ between the combination versus enzalutamide alone (15.2 months vs. 16.6 months, HR 1.12, 95% CI 0.91–1.37, *p* = 0.28). 12.2% Grade 3–5 AEs were reported in the atezolizumab plus enzalutamide cohort versus 1.3% in the enzalutamide cohort.

There are special populations of patients with prostate cancer in whom we may be able to enrich for response to immunotherapies. One of these special population is patients with MSI-H tumors. The US FDA approved pembrolizumab for patients with unresectable or metastatic, MSI-H or mismatch repair-deficient (dMMR) solid tumors in 2017, based on five separate single-cohort studies. Of the 149 patients who were pooled together, two patients had prostate cancer. One patient had a partial response and the second had stable disease [80]. A subsequent study enrolled 233 patients with MSI-H non-colorectal cancers, of whom 6 patients had prostate cancer. Across the cohort, the objective response rate was 34.3% and median duration of response had not been reached, with majority of responders (78%) having responses greater than 2 years [81]. Given this efficacy across tumor types, patients with metastatic prostate cancer are now recommended to undergo testing for MSI-H status, with a prevalence in this population around 3% [82, 83]. Recently published studies further support the efficacy of PD-1 inhibition in patients with MSI-H mCRPC. In a case series of 23 patients with MSI-H/ dMMR mCRPC, 11 patients were treated with anti-PD-1/PD-L1 therapy and 6 patients (54.4%) achieved a ≥ 50% PSA decline. Radiographic responses occurred in 4 out of these 6 patients, and 5 patients were still on therapy for as long as 89 weeks [83]. Graham et al. recently reported that of a total of 17 patients with dMMR and/or MSI-H mPC who received pembrolizumab, 53% had a ≥ 50% PSA reduction, and 87.5% of them remained on treatment at a median follow-up of 12 months [84]. Taking into account the above findings, microsatellite testing should be undertaken and pembrolizumab considered for patients with MSI-H status.

Through a downstream impact on modulation of DNA repair pathways and hence genomic instability, those patients with mCRPC who harbor cyclin-dependent kinase 12 (*CDK12*) loss appear to respond well to immune checkpoint inhibition. CDK12 was shown to phosphorylate RNA polymerase, contributing to homologous recombination repair. With biallelic *CDK12* loss, homologous recombination repair is impaired, inducing an immunogenic subtype of mCRPC with elevated neoantigen burden, increased T‐cell infiltration and clonal expansion. Two of four patients who had *CDK12* loss had significant PSA responses to anti-PD1 monotherapy [85]. According to a recently published multicenter retrospective study of 52 patients with *CDK12*-mutated prostate cancer, at a median follow-up of 8.2 years (95% CI 5.6–11.1), 49 of 52 (94%) patients developed metastatic disease. The median OS from metastasis was 3.9 years (95% CI 3.2–8.1). For the 19 patients treated with any ICI, the > 50% PSA decline rate was 11%, and the estimated 9-month PFS was 23% [86]. Another retrospective multicenter study identified 60 patients with at least monoallelic *CDK12* alterations. In this series, nine patients who had *CDK12* alterations were treated with either pembrolizumab or nivolumab, of whom 33.3% (3/9) had a PSA response and a median PFS of 5.4 months [87]. This suggests *CDK12* deficiency contributes to impaired HRR and has been shown to associate with immunotherapy response.

Completed and ongoing clinical trials investigating different ICI agents in patients with mPC have been summarized (Table 3). Although monotherapy ICIs have not been successful, there are many ongoing trials to combine ICIs with standard chemotherapies or targeted therapies in order to improve clinical outcomes.

**Table 3:** Ongoing clinical trials investigating the administration of immune checkpoint inhibitor agents in patients with mPC

Mechanism	Agent	Concurrent administered agent (Clinicaltrials.gov identifier)	Clinicaltrials.gov identifier	Other ongoing trials (number of subjects)
Anti-PD1	Nivolumab	1. Ipilimumab (followed by nivolumab maintenance therapy)	1. NCT03570619-IMPACT	NCT03835533 (45) NCT03600350 (41) NCT02933255 (29) NCT02601014 (15)
		2. Rucaparib, docetaxel, or enzalutamide	2. NCT03338790-Checkmate 9KD	
	Pembrolizumab	1. Docetaxel	1. NCT03834506-KEYNOTE-921	NCT02499835 (72)
		2. Enzalutamide	2. NCT03834493-KEYNOTE-641	NCT04090528 (60)
		3. Olaparib	3. NCT03834519-KEYLYNK-010	NCT02325557 (51)
		4. (a) olaparib, (b) docetaxel + prednisone, (c) enzalutamide, (d) abiraterone + prednisone	4. NCT02861573/KEYNOTE-365	NCT03093428 (45)
Anti-PDL1	Atezolizumab	Enzalutamide	NCT03016312-IMbassador250	NCT02655822 (336)
Anti-PDL1 + anti-CTLA-4	Durvalumab + tremelimumab	Tremelimumab (IV)	1. NCT03204812	NCT02484404 (384)
		2. Tremelimumab (vaccine) plus PolyICLC	2. NCT02643303	
Anti-CTLA4	Ipilimumab	1. ADT	1. NCT01377389	
		2. Abiraterone acetate + prednisone	2. NCT01688492	

### Other immunotherapeutic targets

Toll-like receptor (TLR)-3 is a pattern recognition receptor expressed on DCs. After binding to double-stranded ribonucleic acid (dsRNA) produced by virus-infected cells, DC-mediated cytokine release can activate TAA-specific CTLs. TLR-3 activation serves as an adjunct to the priming of CTLs [88, 89]; multiple ongoing trials are investigating the combination of TLR-3 agonist poly-ICLC (an analogue of viral dsRNA) with an ICI agent to produce a more robust immune response in patients with mPC (NCT02643303, NCT03835533).

**Fig. 1:**
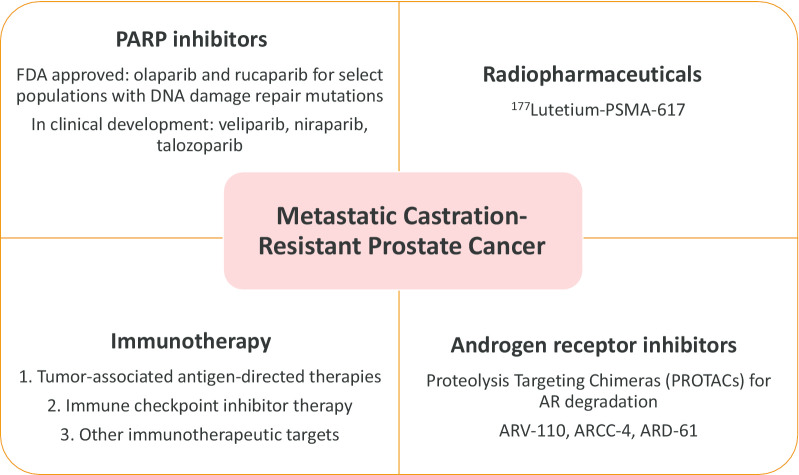
Summary of novel therapeutic categories in metastatic castration-resistant prostate cancer

The adenosine signaling pathway is another attractive target for further investigation in oncology. The adenosine 2A receptor (A2AR) is expressed on a wide variety of immune cells (particularly T cells) [89, 90]. Inhibiting or knocking out A2AR in preclinical models led to tumor regression, spurring the development of the oral A2AR antagonist CPI-444 (ciforadenant), currently in a phase 1/1b trial of 336 patients, alone and in combination with atezolizumab in the treatment of various metastatic solid tumors, including mCRPC (NCT02655822). Another A2AR antagonist, AZD4635, is also currently in development, with an ongoing phase-2 trial in combination with either durvalumab or oleclumab for treatment of mCRPC (NCT04089553). These novel therapeutic strategies for mCRPC may expand future treatment options in this aggressive and terminal disease state (Fig. 1).

### Emerging strategies for the discovery of novel therapeutics in mCRPC:

Novel targets and treatment options cannot be developed without new preclinical models and platforms to find new mechanisms of resistance and targets for future treatments. Metastatic castrate-resistant prostate cancer (mCRPC) patient-derived xenografts (PDXs) recapitulate the genetic and phenotypic diversity of the disease. According to recently published data, LuCaP PDX/organoid models provide an expansive, genetically characterized platform to evaluate mechanisms of pathogenesis as well as therapeutic responses and their molecular correlates in mCRPC [91]. Furthermore, the application of a focused CRISPR-Cas9 screen showed that the concurrent inhibition of RNAP2 and RBX1 profoundly suppresses the growth of CRPC in a synergistic manner, which potentiates the therapeutic efficacy of the RNAP2 inhibitor, α-amanitin-based antibody drug conjugate (ADC) [92]. Moreover, the development of another novel CRISPR-mediated knock-in cell line has showed that PARP inhibitors down-regulate AR signaling and concurrently attenuate androgenic cell growth and promote ‘BRCAness’ to sensitize cells to DNA-damaging agents [93]. In parallel, omics-driven potential drug targets have been evaluated in preclinical models and even in clinical trials, holding promising therapeutic treatment options in patients with advanced PC [94].

## Conclusions

Patients with mCRPC eventually have disease progression on cytotoxic chemotherapy and androgen-axis-targeting drugs, contributing to an ongoing clinical need for novel treatment approaches. The recently US FDA-approved PARP inhibitors, olaparib and rucaparib, have emerged as a treatment option in patients with mCRPC and HRR mutations. In addition, recent results suggest that ^177^Lu-PSMA may offer a potentially effective therapeutic option in patients with mCRPC and high PSMA expression. Patient selection by molecular and genetic markers also offers potential utility for various immunotherapies in mCRPC. Other classes of novel treatments such as AR degraders are still in clinical development. Further clinical development of these novel treatment agents, either alone or in combination with prostate-cancer targeting therapies, will be essential to frame optimal management strategies for this challenging disease. The landscape of treatment options thus continues to expand for patients with mCRPC.

## Data Availability

Not applicable.

## Abbreviations

PC: Prostate cancer
mCRPC: Metastatic castration-resistant prostate cancer
PARP: Poly ADP-ribose polymerase
mPC: Metastatic prostate cancer
ADT: Androgen deprivation therapy
HSPC: Hormone-sensitive prostate cancer
CRPC: Castration-resistant prostate cancer
US FDA: United States Food and Drug Administration
SSBs: Single-strand DNA breaks
DSBs: Double-strand breaks
HRR: Homologous recombination repair
NHEJ: Non-homologous end-joining
PFS: Progression-free survival
OS: Overall survival
OR: Objective response
PSA: Prostate-specific antigen
rPFS: Radiographic PFS
ORR: Objective response rate
AEs: Adverse events
AR: Androgen receptor
nmCRPC: Non-metastatic CRPC
MFS: Metastasis-free survival
PSMA: Prostate-specific membrane antigen
PET: Positron emission tomography
RCT: Randomized controlled trial
CTLs: Cytotoxic T lymphocytes
TAAs: Tumor-associated antigens
mAbs: Monoclonal antibodies
DCs: Dendritic cells
ICI: Immune checkpoint inhibitor
TMB: Tumor mutational burden
MHC-1: Major histocompatibility complex-1
CTLA-4: Cytotoxic T lymphocyte-associated protein-4
PD-1: Programmed death-1 inhibitor
MSI-H: Microsatellite instable-high
DOR: Duration of response
dMMR: Mismatch repair deficient
TLR-3: Toll-like receptor
A2AR: Adenosine 2A receptor
PDXs: Patient-derived xenografts
